# Genome of an *Escherichia coli* engineered for improved control over polyhydroxyalkanoate monomer composition

**DOI:** 10.1128/mra.01423-25

**Published:** 2026-03-16

**Authors:** Robert E. Mejia, Patricia Q. Tran, Deepak Kumar, Brian G. Fox, Erica L-W. Majumder

**Affiliations:** 1 Department of Biochemistry, University of Wisconsin-Madison 5228, Madison, Wisconsin, USA; 2 Department of Bacteriology, University of Wisconsin-Madison 5228, Madison, Wisconsin, USA; 3 Department of Chemical Engineering, SUNY College of Environmental Science and Forestry 14797, Syracuse, New York, USA; University of Maryland School of Medicine, Baltimore, Maryland, USA

**Keywords:** polyhydroxyalkanoate, *Escherichia coli*, metabolic engineering

## Abstract

*Escherichia coli* LSBJ is a strain engineered to produce polyhydroxyalkanoate (PHA) from diverse carbon sources with enhanced control of PHA composition. We report a high-quality annotated genome with 98.68% completeness, 4,547 coding sequences, 75 tRNAs, and 1 copy each of the 5S, 16S, and 23S ribosomal RNA sequences.

## ANNOUNCEMENT

*Escherichia coli* is a foundational microbial host valued globally for its rapid growth rate and genetic tractability. To generate *E. coli* LSBJ, a polyhydroxyalkanoate (PHA) production strain, two beta-oxidation pathway genes—the S-enantiomer-specific enoyl-CoA hydratase *fadB* and its homolog *fadJ*—were deleted from the chromosome of *E. coli* LS5218 (1, 2). These mutations shunt the enoyl-CoA intermediates of beta-oxidation into PHA production (1). In combination, constitutive expression of fatty acid uptake transporters and re-routing of beta-oxidation provide enhanced control of PHA monomer composition. The engineered *E. coli* LSBJ is an efficient producer of medium-chain length PHAs (1, 3). The availability of a high-quality annotated genome provides a foundation and blueprint for further metabolic engineering of PHA production.

*E. coli* LSBJ was received as a gift from the Nomura Lab at the State University of New York College of Environmental Science and Forestry in 2019. It was stored as a glycerol stock at −80°C. *E. coli* LSBJ was plated on a luria-broth (LB) agar and grown at 37°C. Ten milliliters of LB was inoculated with a single *E. coli* LSBJ colony and incubated at 37°C overnight. Genomic DNA was subsequently purified using the Genomic DNA Purification Kit (Thermo Scientific cat. K0512). Sequencing libraries were prepared and sequenced by SeqCenter (Pittsburgh, PA, USA). Library preparation was done using the tagmentation-based and PCR-based Illumina DNA Prep Kit (Illumina cat. 20060060) and custom IDT 10 bp unique dual indices with a target insert size of 280 bp. The DNA was sequenced on an Illumina NovaSeq X Plus sequencer, yielding a total of 2,972,984 paired-end reads (2 × 151 bp) with 96× sequencing depth. Demultiplexing, quality control, and adapter trimming were performed with bcl-convert (v4.1.5). The reads were assembled using the SPAdes Genome Assembly Tool (v4.0.0) with a k-mer value of 95 (4). Assembly quality was assessed using Quast (v5.3.0), which reported an N50 value of 159,262 and an N90 value of 40,947 (5). After filtering for contigs larger than 500 bp, the final assembly consisted of 73 contigs with the largest at 299,436 bp. The total genome length was 4.7 Mbp with a GC content of 50.74%, an L50 of 11, and an L90 of 30. Contig 44 was identified as the pBBRSTQKAB plasmid, which harbors the PHA synthesis pathway (3). This 9,990 bp plasmid contig encodes eight CDS and has a GC content of 61.2%. The genome was then annotated using the NCBI Prokaryotic Genome Annotation Pipeline (pgap_2025-05-06.build7983) (6). This analysis reported 4,547 coding sequences, 75 tRNAs, and 1 complete copy each of the ribosomal RNA subunits (5S, 16S, and 23S). Coding density was calculated to be 85.7%. Final completeness validation using CheckM (v1.2) confirmed the high quality of the final genome, reporting 98.68% completeness and only 0.15% contamination (7). The taxonomic classification was confirmed as an *E. coli* species using GTDB-Tk (v2.4.0) with the Genome Taxonomy Database (v220) (8). Default parameters were used in all steps except where otherwise noted.

## Data Availability

This Whole Genome Shotgun project is deposited at DDBJ/ENA/GenBank under the accession number JBSSVH000000000, and the version described here is JBSSVH010000000. The raw Illumina reads can be found under SRA run accession number SRR36204707. This genome project is indexed at GenBank under BioProject PRJNA1370040, and the microbe sample information can be found under BioSample SAMN53415128.
